# Methodological advancements in organ-specific ectopic lipid quantitative characterization: Effects of high fat diet on muscle and liver intracellular lipids

**DOI:** 10.1016/j.molmet.2023.101669

**Published:** 2023-01-12

**Authors:** Dogan Grepper, Cassandra Tabasso, Axel K.F. Aguettaz, Adrien Martinotti, Ammar Ebrahimi, Sylviane Lagarrigue, Francesca Amati

**Affiliations:** 1 Aging and Muscle Metabolism Lab, Department of Biomedical Sciences, Faculty of Biology and Medicine, University of Lausanne, Bugnon 7, Lausanne, Switzerland; 2 Service of Endocrinology, Diabetes and Metabolism, Lausanne University Hospital and University of Lausanne, Lausanne, Switzerland

**Keywords:** Lipid droplets, Intrahepatic lipids, Intramyocellular lipids, Fatty liver, Lipid metabolism, Obesity, Zebrafish

## Abstract

**Objective:**

Ectopic lipid accumulation is a hallmark of metabolic diseases, linking obesity to non-alcoholic fatty liver disease, insulin resistance and diabetes. The use of zebrafish as a model of obesity and diabetes is raising due to the conserved properties of fat metabolism between humans and zebrafish, the homologous genes regulating lipid uptake and transport, the implementation of the ‘3R’s principle and their cost-effectiveness. To date, a method allowing the conservation of lipid droplets (LDs) and organs in zebrafish larvae to image ectopic lipids is not available. Our objectives were to develop a novel methodology to quantitatively evaluate organ-specific LDs, in skeletal muscle and liver, in response to a nutritional perturbation.

**Methods:**

We developed a novel embedding and cryosectioning protocol allowing the conservation of LDs and organs in zebrafish larvae. To establish the quantitative measures, we used a three-arm parallel nutritional intervention design. Zebrafish larvae were fed a control diet containing 14% of nutritional fat or two high fat diets (HFDs) containing 25 and 36% of dietary fats. In muscle and liver, LDs were characterized using immunofluorescence confocal microscopy. In liver, intrahepatocellular lipids were discriminated from intrasinusoid lipids. To complete liver characteristics, fibrosis was identified with Masson’s Trichrome staining. Finally, to confirm the conservation and effect of HFD, molecular players of fat metabolism were evaluated by RT-qPCR.

**Results:**

The cryosections obtained after setting up the embedding and cryopreservation method were of high quality, preserving tissue morphology and allowing the visualization of ectopic lipids. Both HFDs were obesogenic, without modifying larvae survival or development. Neutral lipid content increased with time and augmented dietary fat. Intramuscular LD volume density increased and was explained by an increase in LDs size but not in numbers. Intrahepatocellular LD volume density increased and was explained by an increased number of LDs, not by their increased size. Sinusoid area and lipid content were both increased. Hepatic fibrosis appeared with both HFDs. We observed alterations in the expression of genes associated with LD coating proteins, LD dynamics, lipogenesis, lipolysis and fatty acid oxidation.

**Conclusions:**

In this study, we propose a reproducible and fast method to image zebrafish larvae without losing LD quality and organ morphology. We demonstrate the impact of HFD on LD characteristics in liver and skeletal muscle accompanied by alterations of key players of fat metabolism. Our observations confirm the evolutionarily conserved mechanisms in lipid metabolism and reveal organ specific adaptations. The methodological advancements proposed in this work open the doors to study organelle adaptations in obesity and diabetes related research such as lipotoxicity, organelle contacts and specific lipid depositions.

## Highlights

• Novel step-by-step cryosection allow quantitative lipid droplet characterization.
• Organ-specific ectopic lipids responses to perturbation are measured simultaneously.
• With HFD intramyocellular lipid droplets increase in volume. Intrahepatic lipid droplets increase on numbers.
• Intrahepatocellular lipids are differentiated from circulating lipids in sinusoids.
• This methodological advancement provides exciting path to explore nutritional perturbations in ectopic lipids associated metabolic dysfunctions.

## Graphical abstract

**Figure:**
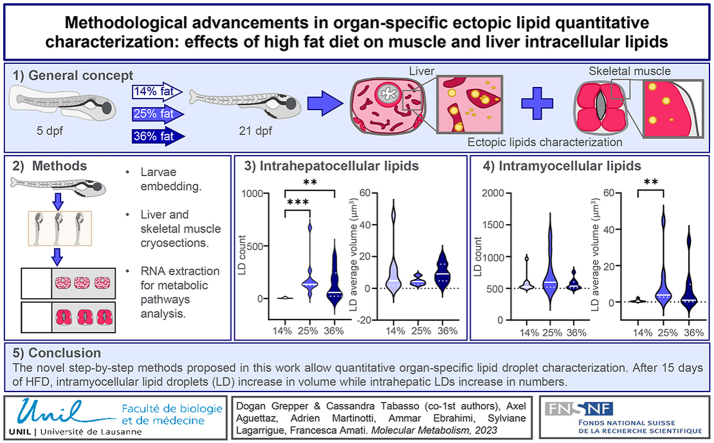

## Abbreviations

*acsl3*: Acyl-CoA synthetase 3
*atgl*: Adipose triglyceride lipase
BSA: Bovine serum albumin
*cidec*: Cell death inducing DFFA like effector C
*cpt1*: Carnitine O-palmitoyltransferase 1
DAG: Diacylglycerol
*dgat2*: Diglyceride acyltransferase 2
Dpf: Days post fertilization
*ef1α*: Alpha subunit of the elongation factor-1 complex
ER: Endoplasmic reticulum
*fabp11a*: Fatty acid binding protein 11a
*faf2*: Fas associated factor family member 2
*gpat4*: Glycerol-3-phosphate acyltransferase 4
HFD: High fat diet
*hsl*: Hormone-sensitive lipase
IHCL: Intrahepatocellular lipids
IMCL: Intramyocellular lipids
LD: Lipid droplet
*ldah*: Lipid droplet associated hydrolase
*lpl*: Lipoprotein lipase
*mettl7a*: Methyltransferase like 7A
*mgl*: Monoacylglycerol lipase
NAFLD: Nonalcoholic fatty liver disease
OCT: Optimal cutting temperature compound
ORO: Oil red O
PBS: Phosphate-buffered saline
PFA: Paraformaldehyde
*plin*: Perilipin
*ppar*: Peroxisome proliferator-activated receptor
RT: Room temperature
SEM: Standard error of the mean
TAG: Triacylglycerol

## 1 Introduction

Excessive fat intake leads to increased lipid storage and adipose tissue overload, consequently causing ectopic lipid depositions and obesity [[1], [2], [3]]. Ectopic lipids are stored in dynamic cytosolic lipid droplets (LDs). In skeletal muscle, ectopic lipid stores are called intramyocellular lipids (IMCL) [2]. IMCL increase along with body fat mass during the development of obesity [4,5]. In the absence of physical activity, IMCL are involved in lipotoxic effects which are associated with altered mitochondrial fatty acid oxidation, apoptosis, inflammation and impaired muscle fiber contractility [6,7]. In the liver, ectopic lipid depositions are called intrahepatocellular lipids (IHCL) [2]. Under physiological conditions, only small amounts of IHCL are stored while most lipids are distributed in the blood stream according to metabolic demand. Hepatic LD accumulation is the hallmark of nonalcoholic fatty liver disease (NAFLD) [8]. In order to investigate human metabolic diseases [9], ectopic lipid depositions in liver and skeletal muscle have been studied in humans and animal models, as well as in vitro in organ specific cells [[9], [10], [11], [12], [13], [14], [15], [16]].

The use of zebrafish as a model to investigate metabolic diseases, particularly obesity and diabetes, is attractive due to the functional conservation of lipid and glucose metabolism as well as the presence of the main organs playing key roles in metabolism (pancreas, muscle, liver, etc) [9]. Compared to other models, zebrafish have similar white adipose tissue depots, such as visceral and subcutaneous adipose tissue [17]. Because zebrafish are ectotherms, they lack brown adipose tissue [18]. While previous studies visualized hepatic steatosis [6,19], to our knowledge, the development of subcellular lipid depositions in zebrafish larvae have not been reported. Our aim was to develop the quantitative tools necessary to assess the effect of nutritional perturbations on IMCL and IHCL in zebrafish larvae.

Organ specific characterization of LDs requires adjusted protocols for embedding, sectioning and visualization [20] depending on samples and organisms. While cryosection preparation has been optimized for portions of adult zebrafish [21], larvae require full body embedding for cryosection due to their small size and heterogeneous texture. Paraffin and resin embedding techniques described previously are either unsuitable to study lipids due to alcohol dehydration [22] or expensive and time consuming [23]. Here, we custom-made a cryosection and imaging procedure to visualize and characterize tissue specific LDs in zebrafish larvae.

## 2 Methods

### 2.1 Zebrafish husbandry

Zebrafish (*Danio rerio*, Oregon AB) were housed at the Zebrafish facility of the Faculty of Biology and Medicine, maintained at 28.5 °C on a 14 h light and 10 h dark cycle. Embryos were staged according to days post fertilization (dpf) as previously described [24]. Animal experimentation and husbandry were approved by the *Service de la consommation et des affaires vétérinaires* (SCAV) of the Canton of Vaud.

### 2.2 Custom mold

**Figure 1:**
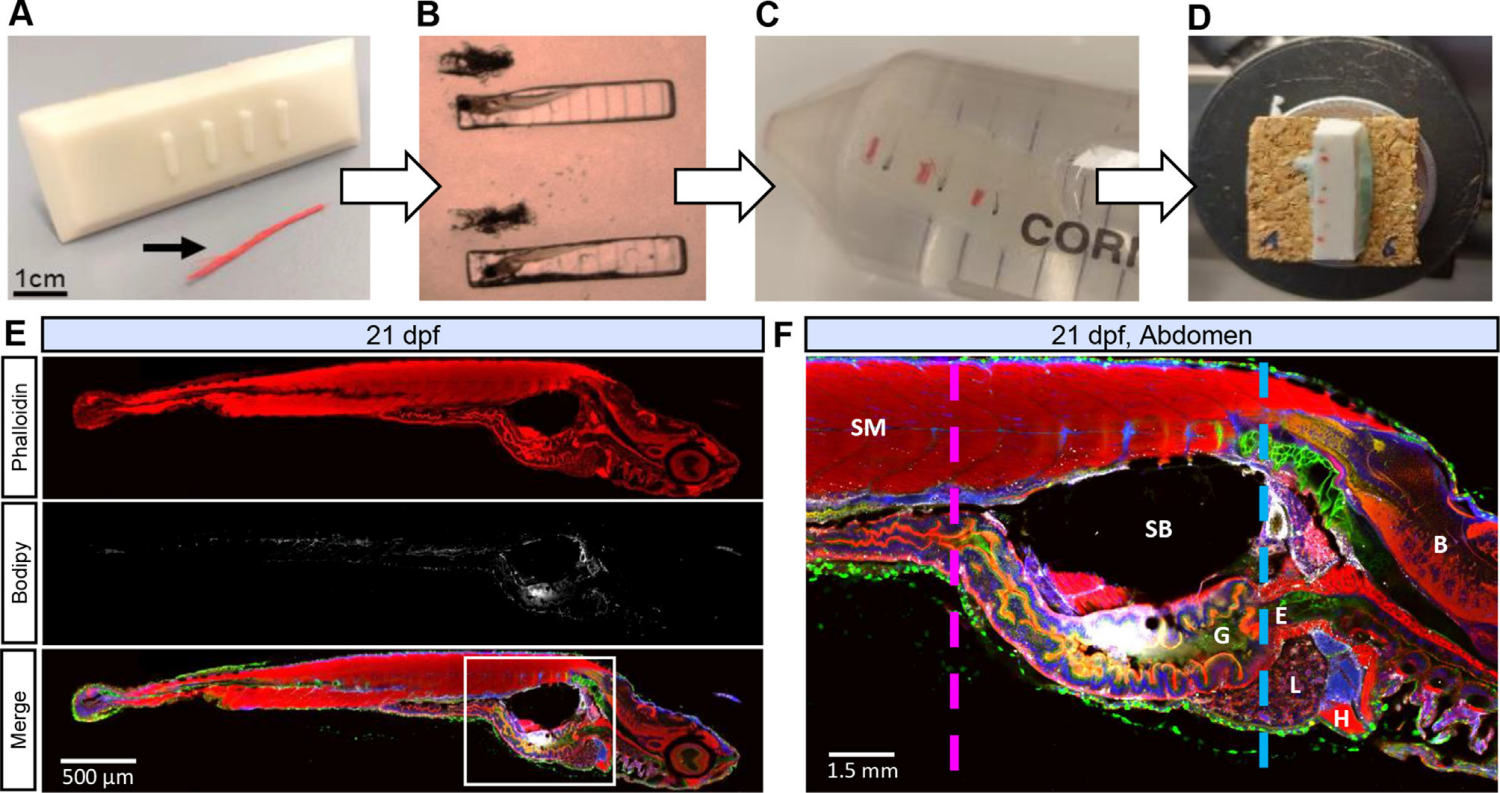
**Stepwise larvae embedding, cryopreservation and cryosectioning. (A)** Custom mold used to form imprints in embedding medium and red waxed dental floss used to mark region of interest (black arrowhead). For exact dimensions, see supplemental data. (**B)** Fixed whole larvae are aligned in imprints. (**C)** After covering with embedding medium, blocks are shaped and cryoprotected with sucrose. (**D)** Blocks are frozen in liquid nitrogen and cut with the cryostat. (**E)** Representative sagittal section of 21 dpf larva fed with normal diet (14% fat diet). (**F)** Close view of the abdomen with muscle (pink dashed line) and liver landmarks (blue dashed line). B: Brain, E: Esophagus, G: Gut, H: Heart, L: Liver, SB: Swim Bladder, SM: Skeletal Muscle. Actin labeled with phalloidin (red), cell membrane labeled with WGA (green), LDs stained with Bodipy665 (white), nuclei stained with Hoechst (blue).

To control larvae position in the histology blocks, we designed 3 custom molds (modified from Sabaliauskas *et* al. [25]) to create imprints adapted for each larval age (Figure 1A). The molds were 3D printed by a professional service (FabArt3D, https://www.fabart3d.ch/, Montreux, Switzerland) with digital light processing resin (PrimaCreator, Malmö, Sweden). Details of our custom molds are available in the supplementary data.

### 2.3 Embedding and cryosections

Embedding medium was prepared by boiling 10 g Type B bovine skin gelatin (Sigma, Kawasaki, Japan), 5 g sucrose (Sigma) and 1.5 g agarose (Roche, Basel, Switzerland) in 100 ml of phosphate-buffered saline (PBS, Dr. G. Bichsel AG, Interlaken, Switzerland) (modified from Nelson *et* al. [21]). The medium was poured warm into petri dishes with the molds placed on top and removed once the medium cooled down enough. Nine 21 dpf larvae per condition were added one by one into the imprints oriented with the head on the deeper side (Figure 1B). To mark the region of interest, red waxed dental floss (Johnson & Johnson, New Brunswick, New Jersey) (Figure 1A and B) was inserted into the medium beside each larva, which were then covered by the liquid medium once it cooled below 50 °C. Once solid, each block was shaped distinctly and put in 0.9 M sucrose overnight at 4 °C (Figure 1C). The blocks were placed in isopentane (Sigma–Aldrich, Saint-Louis, USA) cooled with liquid nitrogen for 2–3 min, and then placed in liquid nitrogen. Each block was mounted on a small piece of cork (Figure 1D) with optimal cutting temperature compound (OCT) (Thermo Fisher Scientific, Waltham, USA). Serial sections of 10 and 30 μm were performed in a cryostat (CM3050S, Leica Microsystems, Wetzlar, Germany) at −18 °C and placed on precleaned glass slides (Thermo Fisher Scientific). Sections were performed for the entire volume where the red string was visible in the blocks. Timings for each procedure are detailed in the supplemental Table 1.

### 2.4 Experimental design

**Figure 2:**
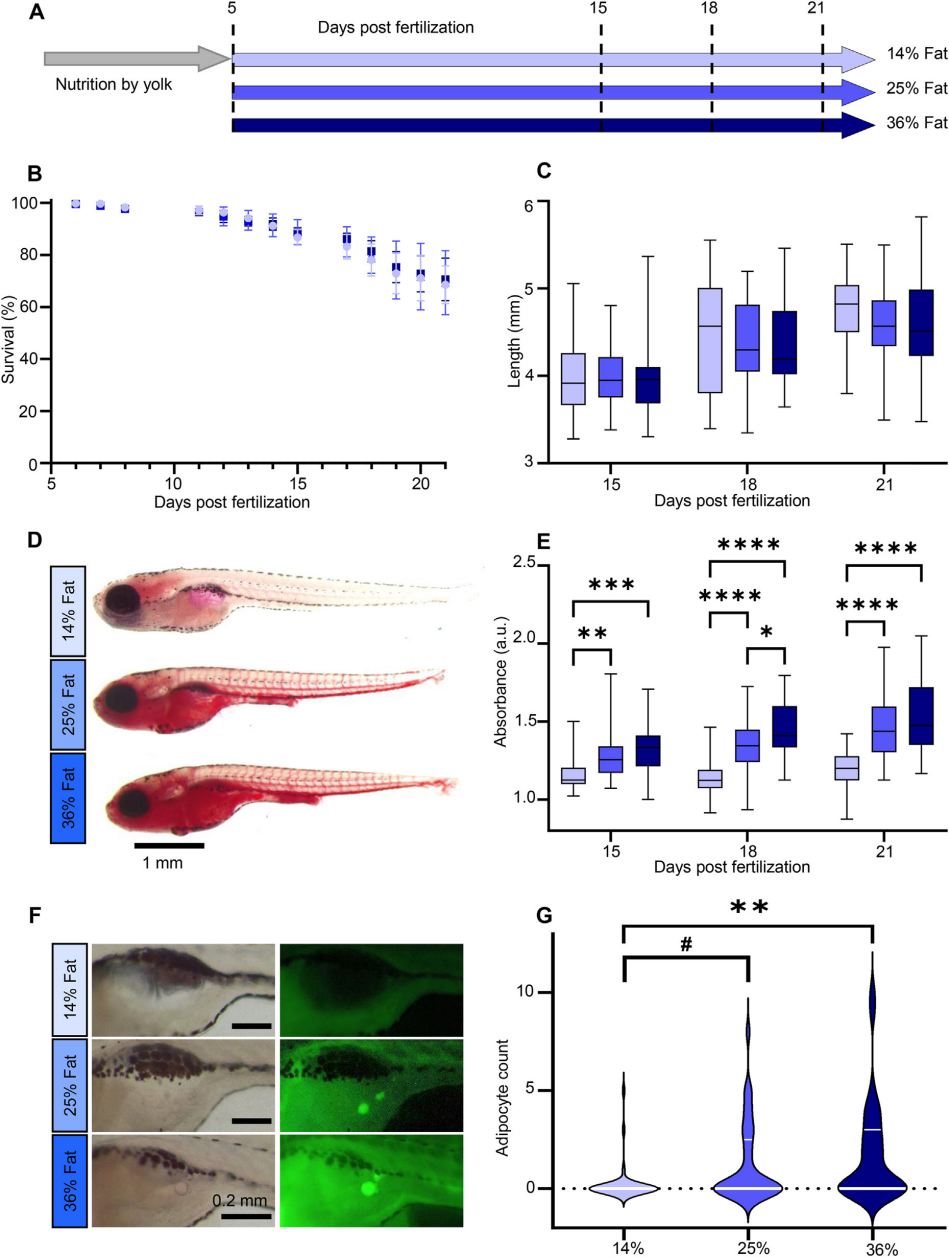
**Study design and high fat diets validation. (A)** Experimental design. Three cohorts of 50 larvae per group were studied at 15, 18 and 21 dpf. **(B)** Survival curve. Bars represent mean ± SEM. Starting n = 250 in three independent cohorts (3 × 3 ANOVA main effect of diet (F(2, 111) = 0.499, p = 0.609)). **(C)** Larvae length. Whiskers represent min. to max., n = 36 pooled from three independent cohorts (3 × 3 ANOVA main effect of diet (F(2, 317) = 1.628, p = 0.198)). **(D)** Representative Oil red O (ORO) images of 21 dpf larvae. **(E)** ORO extraction absorbance at 495 nm normalized to unstained samples. Whiskers represent min. to max., n = 36 per diet for each of the three independent experiments. **(F)** Representative Nile Red images of visceral adipocytes at 21 dpf. **(G)** Adipocyte count distribution per diet. Median and 75th quartile are shown by white lines, n = 33 per diet (Kruskal–Wallis test with Dunn’s multiple comparisons correction). For all panels: #p = 0.0525, ∗p < 0.05, ∗∗p ≤ 0.01, ∗∗∗p < 0.001, ∗∗∗∗p < 0.0001.

To allow the implementation and confirmation of the usefulness of our methodological advancement, we used a three parallel arms interventional design (Figure 2A). One cohort of 750 larvae was divided at 5 dpf in three groups of 250 larvae, each group housed in one tank. One group was fed a control diet (14% of fat), one group was fed a HFD with 25% of fat and one group was fed with a HFD with 36% of fat. To ensure reproducibility, three successive cohorts were used as three independent repeats.

All diets contained Zebrafeed <100 μm (Sparos, Olhão, Portugal), a nutritionally balanced microdiet for zebrafish, either alone or complemented with egg yolk (Eigelbpulver, Bouhwouis Enthoven, Bodenhaltung, Austria) to reach the desired level of nutritional fat (Table 1A). All groups were fed 4 meals per day with the same nutritional mass, which was adapted to larval age (Table 1A). Calories and protein content of the three different diets are shown in Table 1B and C.

**Table 1:** Diets.

**(A) Food composition**			
Days post fertilization	14% Fat	25% Fat	36% Fat
5–9	12 mg ZF	9 mg ZF + 3 mg EY	6 mg ZF + 6 mg EY
10–12	16 mg ZF	12 mg ZF + 4 mg EY	8 mg ZF + 8 mg EY
13–21	20 mg ZF	15 mg ZF + 5 mg EY	10 mg ZF + 10 mg EY

**(B) Caloric content**			
Diet type	Calories for 12 mg	16 mg	20 mg
**14% Fat**	12 × 4.8 = **57.6 cal**	**76.8 cal**	**96 cal**
**25% Fat**	9 × 4.8 + 3 × 6.6 = **63 cal**	**84 cal**	**105 cal**
**36% Fat**	6 × 4.8 + 6 × 6.6 = **68.5 cal**	**91.3 cal**	**114 cal**

**(C) Protein content**			
Diet type	Proteins for 12 mg	16 mg	20 mg
**14% Fat**	12 × 60.3% = **7.2 mg**	**9.7 mg**	**12.1 mg**
**25% Fat**	9 × 60.3% + 3 × 15.8% = **5.9 mg**	**7.9 mg**	**9.8 mg**
**36% Fat**	6 × 60.3% + 6 × 15.8% = **4.6 mg**	**6.1 mg**	**7.6 mg**

ZF is Zebrafeed, EY is egg yolk.

Housekeeping included daily cleaning of all tanks and 30% water replacement. An air bubbler ensured uniform oxygenation and equitable access to food despite different textures and floating time. Dead larvae count was performed each morning to assess survival rate.

At 15 dpf, 18 dpf, and 21 dpf, 50 larvae per group were randomly selected and fasted overnight for 16 h. Euthanasia was performed with hypothermic shock followed either by flash freezing and conservation at −80 °C until further usage for gene expression analyses or by immediate fixation in 4% paraformaldehyde (PFA) (Carl Roth GmbH, Karlsruhe, Germany) for 5 h at room temperature (RT) and stored in PBS (Dr. G. Bichsel AG) at +4 °C for all other analyses. For each condition, the number of larvae used for the different experiments and outcomes are specified in Supplemental Fig. 1. All the experiments presented below were conducted in 3 experimental repeats (3 independent cohorts). Unless specified, animal numbers presented in the results correspond to the sum of the 3 repeats.

### 2.5 Larvae length

Brightfield images of 36 fixed larvae per condition and time point were taken with a Leica M165 F-C (Leica Microsystems). Length was measured from the mouth and along the spine until the beginning of the caudal fin using Fiji [26].

### 2.6 Whole larvae neutral lipid content

Oil Red O (ORO) stock solution was prepared by diluting 3% of ORO (Sigma–Aldrich) in 60% isopropanol (Sigma–Aldrich) and filtering it through a 0.22 μm filter (MILLEX GV, Duluth, USA). 36 fixed larvae per condition and time point were stained for 2 h, rinsed three times with 60% isopropanol and 3 times with PBS. Three supplemental larvae from each group served as an unstained control for normalization. Each individual larva was placed in a well of a 96-well plate (Corning, Corning, USA) and ORO was extracted with 80 μl of pure isopropanol per larva for 2 h while being rocked on an oscillator. 60 μl of the total extraction solution were used for absorbance measured with a Synergy Mx microplate reader (Biotek, Winooski, USA) at 495 nm.

### 2.7 Whole larvae adipocyte count

Before euthanasia, 33 larvae per condition were stained in Nile Red (Sigma–Aldrich) diluted in acetone with housing water to a final Nile Red concentration of 0.5 μg/ml [27]. Larvae were then placed in the dark for 30 min, washed twice with housing water, sacrificed and fixed in 4% PFA for 5 h at RT. Brightfield and fluorescence images were acquired with a Leica M165 F-C fluorescence stereomicroscope (Leica Microsystems). Adipocytes were counted using Fiji.

### 2.8 Immunostaining

Slides with 30 μm sections from nine larvae per condition were fixed in 4% PFA for 15 min at RT. Sections were washed thrice with PBS and permeabilized with 0.5% Triton (Sigma–Aldrich), washed and blocked with 1% PBS-BSA (bovine serum albumin) (Sigma). Staining was performed with a combination of Bodipy665 (D10001, Thermo Fisher Scientific) diluted at 1/500, Phalloidin (SC-363797, Santa Cruz Biotechnology, Dallas, USA) diluted at 1/2000, WGA FITC (GTX01502, GeneTex, Irvine, USA) at 1/1000, all in PBS-BSA 1% for 2 h at RT then rinsed three times with PBS. Finally, the slides were stained with Hoechst (H3570, Invitrogen Molecular Probes, Eugene, USA) at 1/8000 for 2 min and rinsed thrice. Slides were mounted with Mowiol (Sigma–Aldrich) covered with a 24 × 50 mm coverglass (Merck, Darmstadt, Germany). Images were acquired using a spinning disk confocal microscope (Nikon Ti2, CrEST Optics aX-Light V3, Nikon, Tokyo, Japan) at a 60X magnification in oil immersion (CFI Plan Apochromat Lambda 60X Oil, N.A1.40, W.D. 0.13 mm, Nikon) in stacks of 15 μm thickness with 0.2 μm Z-steps.

### 2.9 Skeletal muscle LD number and volume

Muscle images from nine 21 dpf larvae per condition were deconvolved using a deconvolution matrix in the Huygens Remote Manager (Huygens Software, https://hrm.svi.nl/, Scientific Volume Imaging B.V., Hilversum, The Netherlands). The images were processed using Imaris 9.8 (Oxford Instruments, Abingdon, England) in a blinded fashion. In each muscle quadrant, LDs were reconstructed in one volume of 38 × 38 × 15 μm^3^ (Supplemental Fig. 2). The volume of each LD was then acquired.

### 2.10 Liver LD and sinusoid lipids quantification

Liver images from nine 21 dpf larvae per condition were deconvolved as described above. To quantify LDs in the parenchyma, LDs were reconstructed using Imaris 9.8 in twelve equal volumes (15 × 15 × 10.5 μm^3^) blindly selected and processed as described above. The volume of each LD was acquired.

Sinusoids were defined using the combination of a thick WGA staining to identify the endothelial glycocalyx layer [28,29] and confirmed with actin staining [30]. The Z-stack was used to exclude hepatocytes surrounded by immune cells or stellate cells, ascertaining the absence of subcellular compartments. One 60 × 60 μm^2^ region of interest (ROI) was selected in each blinded liver image. Using Fiji, sinusoid surface was determined and measured. A background of 20 was removed from the Bodipy channel to prevent possible deconvolution artifacts. Machine-learning based segmentation was performed using Ilastik [31]. Bodipy positive area were quantified inside the sinusoids on the obtained binary images (322 × 322 pixels) using Fiji’s analyze particle function. Given the high density of particles in the intrasinusoid space, we chose to measure surface instead of particle size to take into account that chylomicrons were possibly on top of each other or coalescent.

### 2.11 Liver fibrosis

Slides containing 10 μm sections from nine 21 dpf larvae per condition were dried overnight at −20 °C, and stained with modified Masson’s Trichrome without hematoxylin to prevent excessive darkness. Images were acquired with 10X to 40X objectives using an ECLIPSE 90i microscope (Nikon) with a Nikon digital sight camera. For quantification, 40X images were blinded and liver tissue was selected. Macros in Fiji were used to measure liver and fibrotic areas. The percentage of fibrotic tissue was computed as the ratio between the fibrotic area and the parenchyma.

### 2.12 RNA extraction

25 snap frozen larvae per time point per group per cohort were pooled for RNA extraction adapted from guidelines provided by Sigma–Aldrich for TRI Reagent RNA extraction. In brief, larvae were lysed using a mechanical pestle (Kimble Chase, Vineland, USA) in TriReagent (Sigma). RNA was isolated with chloroform (Sigma–Aldrich), precipitated with isopropanol and resuspended. Final RNA concentration was measured using a ND-1000 Spectrophotometer (Thermo Fisher Scientific).

### 2.13 RT-qPCR

One μg RNA per pool was retrotranscribed using GoScript™ Reaction Buffer (Random Primers) and GoScript™ Enzyme Mix (Promega, Madison, USA). Retrotranscription products were mixed with Power SYBR Green PCR Master Mix (Thermo Fisher Scientific) and with 600 nM of forward and reverse primers (Supplemental Table 2). Samples were analyzed with ViiA 7 Real-Time PCR System (Thermo Fisher Scientific) or QuantStudio 5 (Thermo Fisher Scientific). Relative expression of mRNA was estimated using the 2^−ΔΔCT^ method using *ef1α* as reference.

### 2.14 Statistical analyses

Data are presented as mean and standard error of the mean (SEM). Normality was checked with the Shapiro–Wilk test and equality of variance was assessed with the Bartlett’s test and the Brown–Forsythe test. If assumptions were met, data were analyzed with one-way ANOVA and multiple comparisons were performed using Tukey HSD. If assumptions were not met, data were analyzed with the Kruskall–Wallis test and multiple comparisons were performed using the Dunn’s test. Data of the length, survival rate and ORO absorbance were analyzed with a two-way ANOVA with a factor of time (3 levels: 15, 18 and 21 dpf) and of diet (3 levels). All statistical analyses were performed using Prism (GraphPad, version 9.0.0, San Diego, USA) with the significance set at p < 0.05.

## 3 Results

### 3.1 Organ landmarks

As shown in Figure 1E and F, the cryosections obtained after setting up the embedding and cryopreservation method (Figure 1A–D) were of high quality, preserving tissue morphology and allowing the visualization of ectopic lipids. To investigate IMCL, we used skeletal muscle transversal sections at the topological marker of the junction between mid-intestine and intestinal bulb as indicated by the pink dashed line in Figure 1F. To investigate IHCL, we used the end of the esophagus as topological marker for liver transversal sections as indicated by the blue dashed line in Figure 1F.

### 3.2 High fat diet increased neutral lipid content and adipocyte number in zebrafish larvae

As previously described, larvae survival decreased over time (Figure 2B). Our overall mean survival rate at 21 dpf was of 69.46 ± 0.74%, in the range of reported survival rates [32]. Larvae length increased throughout development (Figure 2C) with a significant simple main effect of time (F(2, 317) = 56.91, p < 0.0001). Survival and length were not significantly different across diet groups (Figure 2B and C).

Larvae neutral lipid content increased with time and dietary fat (Figure 2D and E). The two-way ANOVA revealed a statistically significant interaction (F(4, 310) = 2.971, p = 0.0197). Post hoc comparisons of simple effects of diet (F(2, 310) = 69.65, p < 0.0001) are represented in Figure 2E. To determine the optimal time point to evaluate the effect of HFD on ectopic lipids, we compared 18 and 21 dpf neutral lipid content to 15 dpf, which is the known age when LDs begin to expand beyond the visceral cavity [33,34]. We chose 21 dpf as our time point of interest given that neutral lipid content was significantly higher with both HFDs compared to 15 dpf (p < 0.0001). This was not the case when comparing 18 dpf to 15 dpf. At 21 dpf, the pattern of adipocyte distribution confirmed the effect of the two HFDs (Figure 2F and G).

### 3.3 High fat diet induced an increase in LD volume without increasing LD numbers in skeletal muscle

**Figure 3:**
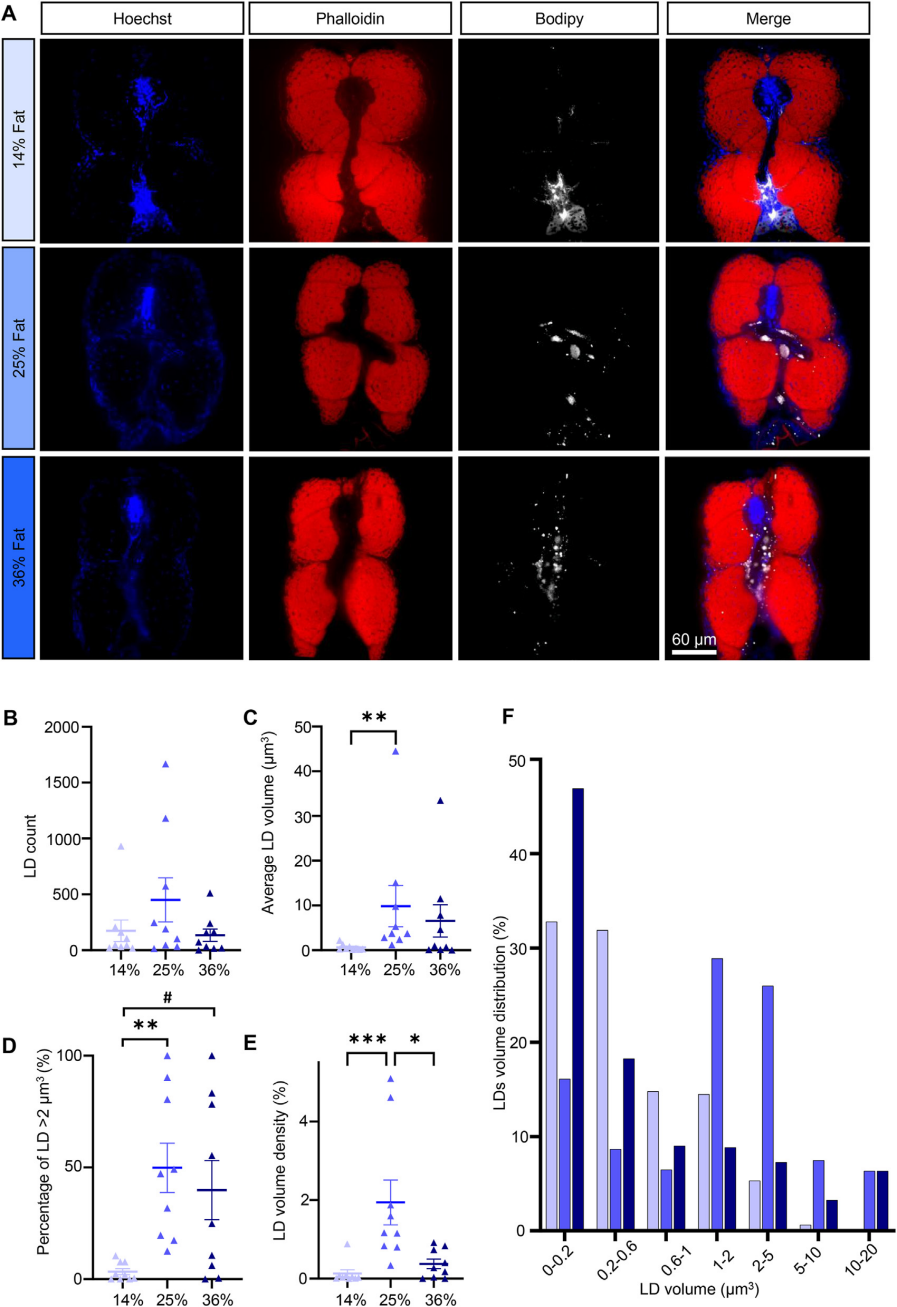
**Intramyocellular lipids. (A)** Representative images of skeletal muscle sections in 21 dpf larvae. Nuclei stained with Hoechst (blue), actin stained with phalloidin (red), LDs stained with Bodipy665 (white). **(B)** LD count per fish. **(C)** Average LD volume per fish. **(D)** Percentage of LDs with a volume >2 μm^3^. **(E)** LD volume density. **(F)** LD volume distribution. Bars represent mean ± SEM, n = 9 pooled from three independent experiments (Kruskal–Wallis test with Dunn’s multiple comparisons correction). For all panels: #p = 0.0586, ∗p < 0.05, ∗∗p ≤ 0.01, ∗∗∗p < 0.001, ∗∗∗∗p < 0.0001.

Intramyocellular average LD volume (Figure 3C, Kruskal–Wallis test p = 0.012) and volume density (Figure 3E, Kruskal–Wallis p = 0.0008) increased with HFD. This was not accompanied by an increase in LD numbers (Figure 3B, Kruskal–Wallis p = 0.383), but by a shift of LD size towards larger LDs (Figure 3F and Supplemental Fig. 2). The amount of LD with a volume above 2 μm^3^ was increased (Figure 3D, Kruskal–Wallis p = 0.0022).

### 3.4 High fat diet increased cytosolic LD numbers, without impacting LD volume in liver, and induced fibrosis

**Figure 4:**
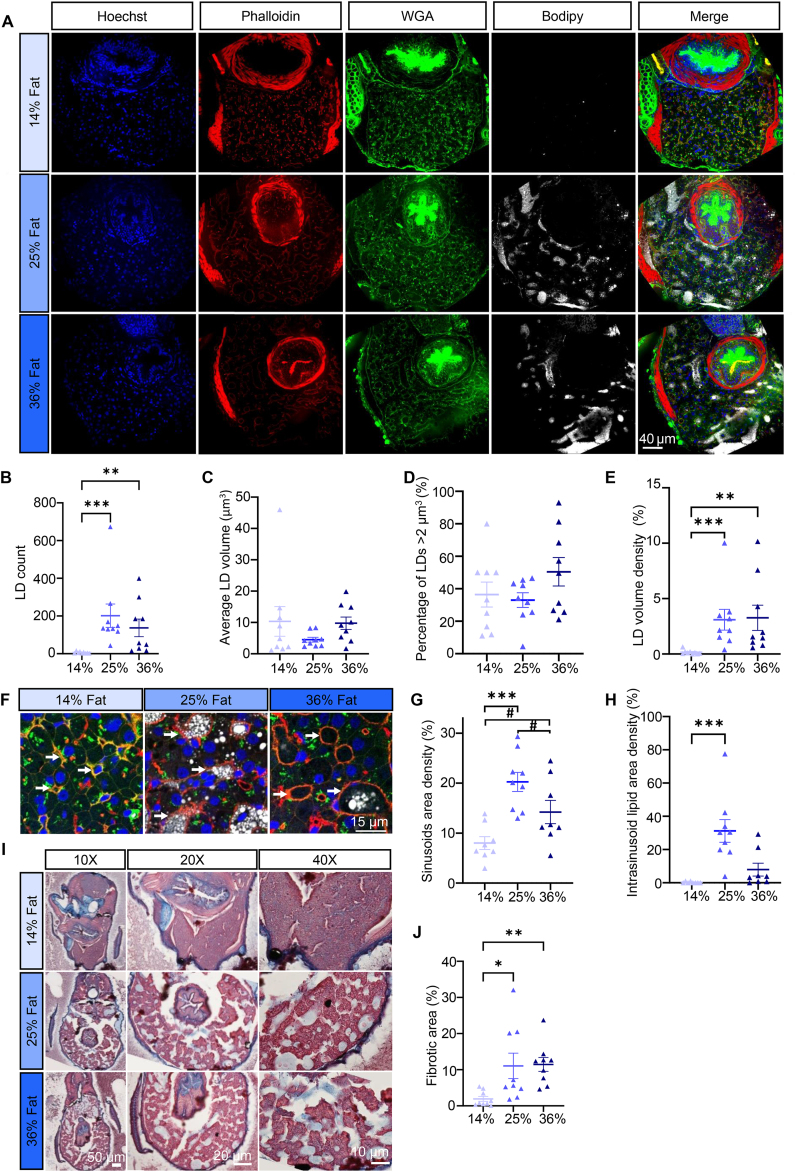
**Intrahepatocellular lipids and fibrosis. (A)** Representative images of liver sections in 21 dpf larvae. Nuclei stained with Hoechst (blue), actin stained with phalloidin (red), cell membranes stained with WGA (green), LDs stained with Bodipy665 (white). (**B)** LD count per fish. (**C)** Average LD volume per fish. (**D)** Percentage of LDs with a volume >2 μm^3^. (**E)** LD volume density. (**F)** Representative close view of liver tissue in 21 dpf larvae for each diet, white arrowheads are sinusoids. (**G)** Sinusoids area density. (**H)** Intrasinusoid lipid area density. (**I)** Representative Masson’s Trichrome images for each diet. (**J)** Fibrotic area. Bars represent mean ± SEM, n = 9 pooled from three independent experiments (For D: one-way ANOVA with Tukey HSD, for all others: Kruskal–Wallis test with Dunn’s multiple comparisons). For all panels: #p = 0.082, ∗p < 0.05, ∗∗p ≤ 0.01, ∗∗∗p < 0.001, ∗∗∗∗p < 0.0001.

Intrahepatocellular LDs increased with HFD (Figure 4B, Kruskal–Wallis p = 0.0002), as did LD volume density (Figure 4E, Kruskal–Wallis p = 0.0003). This was not accompanied by an increase in LD volume (Figure 4C and Supplemental Fig. 2, Kruskal–Wallis p = 0.194) or by the percentage of large LDs (Figure 4D, one-way ANOVA F (2, 24) = 1.635, p = 0.216).

Sinusoids area density was increased with HFD (Figure 4F and G, one-way ANOVA F (2, 22) = 10.59, p = 0.0006). Intrasinusoid lipid area density was increased with 25% HFD (Figure 4H, Kruskal–Wallis p = 0.0003). Hepatic fibrosis appeared with HFD (Figure 4I and J, Kruskal–Wallis p = 0.0011).

### 3.5 Lipid droplet associated genes were differentially affected by high fat diet

**Figure 5:**
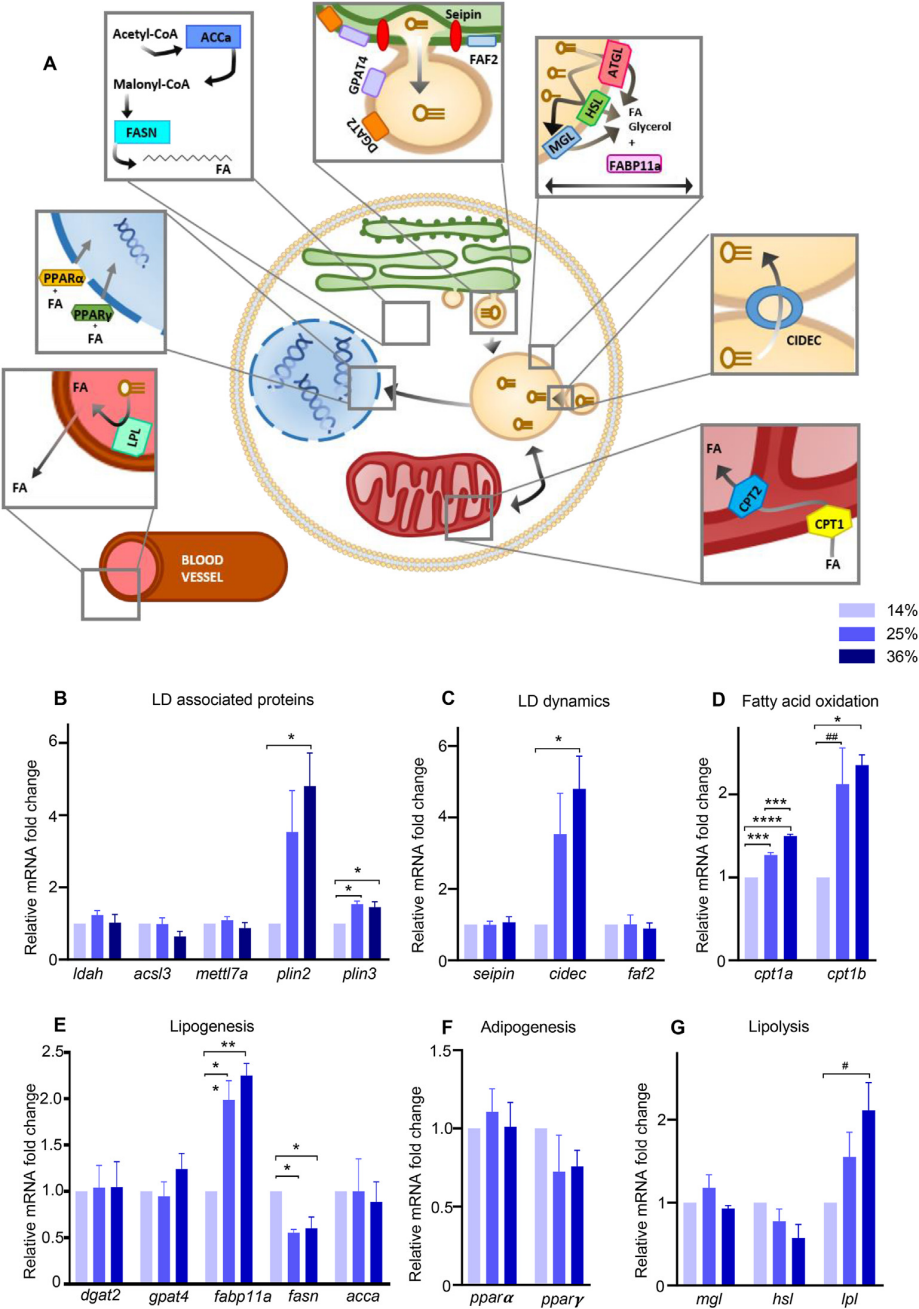
**HFD altered lipid droplets associated pathways. (A)** Schematic representation of target genes involved in LD coating proteins, dynamics, lipolysis, fatty acid oxidation, adipogenesis and lipogenesis. Lipoprotein lipase (*lpl*) hydrolyzes triglycerides and clears circulating lipids in the blood. Peroxisome proliferator-activated receptor α (*pparα*) and *γ* (*pparγ*) are transcription factors controlling lipid metabolism in various tissues. Acetyl-CoA carboxylase alpha (*acca*) catalyzes the carboxylation of acetyl-CoA to malonyl-CoA, which is then synthetized into long chains FA by the fatty acid synthase (*fasn*). Glycerol-3-phosphate acyltransferase 4 (*gpat4*) relocalizes from the endoplasmic reticulum (ER) to forming LDs and catalyzes the first step of triacylglycerol (TAG) synthesis. Diglyceride acyltransferase 2 (*dgat2*) is responsible for the conversion of diacylglycerol (DAG) to TAG. Fas associated factor family member 2 (*faf2*) and seipin are ER proteins which form budding LDs. Adipose triglyceride lipase (*atgl*), hormone-sensitive lipase (*hsl*), and monoacylglycerol lipase (*mgl*) are lipases which breakdown triglycerides stored in LDs. Fatty acid binding protein 11a (*fabp11a*) is involved in lipid transport, storage and breakdown. Cell death Inducing DFFA like effector c (*cidec*) is involved in LD fusion and growth as well as apoptosis, while perilipin 2 and 3 (*plin*), acyl-CoA synthetase 3 (*acsl3*), lipid droplet associated hydrolase (*ldah*) and methyltransferase like 7A (*mettl7a*) are recruited to LD during maturation. Carnitine O-palmitoyltransferase 1a (*cpt1a*) and b (*cpt1b*) transform lipids to allow degradation through β-oxidation by mitochondria. (**B-G)** Gene expression measured by RT-qPCR. Data are normalized with the alpha subunit of the elongation factor-1 complex (*ef1α*). Bars represent mean ± SEM, n = 3 pools of 25 larvae from three independent experiments (One way ANOVA with Tukey HSD). For all panels: #p = 0.0513, ##p = 0.0529, ∗p < 0.05, ∗∗p ≤ 0.01, ∗∗∗p < 0.001, ∗∗∗∗p < 0.0001.

Whole larvae transcriptional changes were studied for targeted genes associated with LD coating proteins, LD dynamics, adipogenesis, lipogenesis, lipolysis and fatty acid oxidation (Figure 5A–G). At 21 dpf, *plin2* and *plin3* were increased by HFD (Figure 5B). *Cidec*, *cpt1a* and *cpt1b*, *fasn and fabp11a* also responded to HFD (Figure 5C–G).

## 4 Discussion

Excess fat and caloric intake are cornerstone of the lipotoxicity theory linking obesity to insulin resistance through the accumulation of ectopic lipids, particularly in skeletal muscle and liver. The use of zebrafish as a model to study obesity, lipid metabolism and diabetes is rising [9,35,36]. Our main aim was to develop a detailed protocol to characterize quantitatively intracytosolic ectopic lipids, in liver and skeletal muscle of zebrafish larvae after a nutritional perturbation. Here, we propose five methodological novelties. First, in order to image whole larvae, we optimized cryosection and cryopreservation methods allowing the conservation of lipid droplets. These protocols can also be of use for many other outcomes and models with small fragile organs. The 3D printed molds allow proper positioning of animals, are adapted for age and length, and provide reproducible outputs. The second innovative aspect is that we present multiple ectopic lipid readouts that are all quantitative. Thirdly, we imaged ectopic lipids in multiple organs in one single study and with the exact same treatment. Fourthly, we differentiate liver parenchyma ectopic lipids from lipids in the sinusoids. This, to our knowledge, has not been done previously in zebrafish, mice or human although lipids in sinusoids are not ectopic lipids but circulating lipids. Finally, we describe a complete step by step protocol investigating the effects of high fat diet on zebrafish larvae, which could also be applied to many other treatments such as high glucose/high fructose diets or drug treatments.

To investigate specific ectopic lipids of our two organs of interest, we developed a zebrafish larvae sectioning method. Paraffin embedding, the current gold standard for zebrafish larvae sectioning, requires ethanol dehydration that causes the loss of lipids from tissues and induces morphological changes [22]. In comparison, our method allows complete tissue conservation of small larvae and does not require dehydration, preventing subsequent deformation of the tissues and loss of lipids. Gelatin embedding provides samples useable for both traditional histology techniques, like Masson’s Trichrome, or immunohistochemical staining. We believe that this methodological development will serve multiple other inquiries, such as studying other organs or organelles of interest.

While most of overfeeding and HFD studies in zebrafish explore adiposity or global hepatic lipids [10,37], our method allowed to quantify ectopic lipids distribution and volume, rather than fluorescence intensity or area as described previously [10,19,[38], [39], [40], [41]]. We tested two HFDs (25 and 36% of fat) to validate their suitability for quantitative ectopic lipids characterization. The increase of neutral lipid content at all time points (15, 18 and 21 dpf), measured by single larvae extraction of Oil Red O to reduce the bias caused by measuring fish color, confirmed the obesogenic effect of both HFDs without impairing larvae development and survival. Similarly to other studies supplementing zebrafish larvae diet with different proportions of egg yolk [42,43], the number of larvae showing adipocytes increased with nutritional fat.

In skeletal muscle, 15 days of HFD feeding caused an increase in skeletal muscle LD size but not LD count. The increased LD size following HFD is in accordance with previous studies in rodents [40] and human volunteers [[44], [45], [46]] pointing to the negative association of larger LDs, but not IMCL per se, to insulin sensitivity. From a mechanistic point of view, the observed LD expansion could be due to an increased TAG synthesis, to ripening or to LD coalescence, with the creation of expanding pores between two LDs [47]. Further, LD expansion also affects LD surface tension, impacting LD monolayer curvature and its components [48], including LD coating proteins and access to the lipolysis/lipophagy machineries.

In liver, HFD increased the number of LDs in the hepatic parenchyma, caused larger sinusoid areas with higher lipid content in their lumen and provoked hepatic fibrosis. Importantly, we separated IHCL, i.e. ectopic lipids in the hepatic parenchyma, from lipid micelles in the sinusoids. In the hepatic parenchyma, the average number of LDs and their volume density increased as described in other models [49], but not LD size. Mechanistically, the potential source of fats contributing to fatty liver could come from the esterification of dietary fatty acids provided in excess through the uptake of intestinally derived chylomicron remnants, from lipogenesis derived fats stored in adipose tissue and/or from liver de novo lipogenesis [50]. In the sinusoids, increased lipids reflect the increased presence of lipoproteins due to their role as lipid transporters and their structure similar to those of LDs [8]. In hepatic injury due to excess lipid exposure, hepatic stellate cells surrounding the sinusoids produce collagen and extracellular matrix. In our larvae, we observed an enlargement of sinusoids accompanied by fibrosis in the HFD groups, confirming the development of hepatic injury.

Given the difficulty of isolating organ-specific mRNA from zebrafish larvae due to their small size and fast degradation, we opted to measure whole body gene expression. This is an important limitation as these results should not be extrapolated to organ-specific gene expression in other models. Similarly to another study executed in zebrafish larvae [43], we did not observe changes in adipogenesis markers with HFD. The fact that zebrafish body composition is mostly composed of fat free mass [51] may cover adipose tissue specific pathways modulations. The upregulation of *fabp11a*, zebrafish ortholog of human FABP4 [10], implies that lipid transport pathways are affected by HFD. The increased expression of *cidec*, which has been demonstrated to form a pore-like structure between LDs [52] also speaks in favor of inter-LD lipid transfer. Further, *Cidec* has been shown to control HFD induced fatty liver [52] and its increased expression supports the development of steatosis in larvae. Supporting the hypothesis of chylomicron remnants in liver sinusoids, we observed an upregulation of *lpl*. *Plin2* and *3* expressions increased with HFD, reflecting the higher volume of LDs and the modulation of LD access to lipases [19,53]. We witnessed altered fatty acid oxidation, characterized by higher *cpt1a* and *cpt1b* mRNA expression. The downregulation of *fasn* suggests the suppression of de novo lipogenesis in response to excess dietary fats.

In our results, we observe differences between the two HFDs in causing ectopic lipids depositions. The 25% fat diet induced significant increases of average LD volume and LD volume density in muscle. These modifications were not observed with the 36% fat diet. In liver, both HFDs increased significantly LD count and LD volume density in hepatocytes. While only the 25% fat diet increased intrasinusoid lipid area density, both diets induce similar fibrosis. These organ-specific observations are in contrast with whole larvae outcomes at the same time point. Indeed, overall ORO absorbance at 21 dpf increased similarly with both HFDs. Significant changes in whole larvae mRNA levels were mostly observed after the 36% HFD or similarly with the two HFDs. Only *cpt1a* increased in an incremental pattern between the 25% and 36% nutritional fat. These observations support the hypothesis that fat metabolism may be enhanced in the 36% fat fed animals, thus using more intramyocellular lipids at a given time point. This may be in part secondary to the relative difference in dietary proteins. To note that all of our diets had a protein content above the minimal juveniles requirements estimated by Fernandes et al. [54] although complete nutritional requirements of both adult and larval zebrafish are not yet determined [32,55].

With a thorough organ-specific LD characterization, our study demonstrates the impact of HFD on ectopic lipid depositions in liver and skeletal muscle, as well as the changes of key players in fat metabolism in zebrafish larvae. We propose a reproducible and fast method for imaging zebrafish larvae without sacrificing LD quality or organ morphology. Our findings confirm the evolutionarily conserved mechanisms in lipid metabolism, reveal organ specific adaptations and open the door to studying organelle modifications in obesity and diabetes related research such as lipotoxicity, organelle contacts and specific lipid depositions in this cost-effective animal model.

## Author contributions

Dogan Grepper: Conceptualization, methodology, validation, investigation, visualization, writing –original draft, review and editing. Cassandra Tabasso: Methodology, validation, formal analysis, investigation, data curation, visualization, project administration, writing –original draft, review and editing. Axel Aguettaz: Investigation, writing – review and editing. Adrien Martinotti: Methodology, investigation. Ammar Ebrahimi: Writing – review and editing. Sylviane Lagarrigue: Methodology, supervision, writing – review and editing. Francesca Amati: Conceptualization, methodology, formal analysis, resources, writing – original draft, writing – review and editing, visualization, supervision, project administration, funding acquisition.

## Funding

This project was supported by the 10.13039/100000001Swiss National Science Foundation grant number 310030_188789 (to FA).

## Conflict of Interest

None declared.

## Data Availability

Data will be made available on request.
