# Supplementary material for: Methodological advancements in organ-specific ectopic lipid quantitative characterization: Effects of high fat diet on muscle and liver intracellular lipids
Source: Mol Metab. 2023 Jan 12;68:101669. doi: 10.1016/j.molmet.2023.101669 (PMC9938329; doi:10.1016/j.molmet.2023.101669)
Supplement: Multimedia component 1 [file mmc1.pdf]

SUPPLEMENTAL INFORMATION

Title

Methodological advancements in organ-specific ectopic lipid quantitative characterization: effects of high fat diet on muscle and liver intracellular lipids

Authors

Dogan Grepper[#], Cassandra Tabasso[#], Axel Aguettaz, Adrien Martinotti, Ammar Ebrahimi, Sylviane Lagarrigue, Francesca Amati*

[#] Shared first authorship

* Corresponding author

Content

- Supplemental figure 1: Larvae numbers

- Supplemental figure 2: Representative images of lipid droplets volume quantification

- Supplemental table 1: Timing of experiments for embedding, staining, imaging and analyses

- Supplemental table 2: QPCR primer sequences of target genes

- Supplementary data with custom molds details. The following files are available at https://wwwfbm.unil.ch/dsb/Files_Francesca_Amati.zip :

    small_bedsize_X.jpg

    small_bedsize_Y.jpg

    fishmold_versions.jpg

    fishmold_blend (source file for software Blender)

    shoe_mold_base_SLA_neg_z12_propedit_v2_new_V3_sc25_shx-20.stl (small)

    shoe_mold_base_SLA_neg_z12_propedit_v2_new_V3_sc50_shx-20.stl (medium)

    shoe_mold_base_SLA_neg_z12_propedit_v2_new_V3_shx-20.stl (large)

    manche_new_V2.stl (handle, printed filament deposition FDM)

Supplemental figure 1: Larvae numbers per tank

A) For each nutritional condition, 250 larvae were placed in one tank. The numbers of larvae presented in this pie chart are those randomly selected at each time point. **B)** From the sampled larvae at 21 dpf, the numbers presented in this pie chart are those used for each experiment/outcome. LD means larvae used for fluorescent imaging of LDs in liver and skeletal muscle. Given that 3 independent cohorts were analyzed, total numbers correspond to three times those presented in the pie chart for each condition.

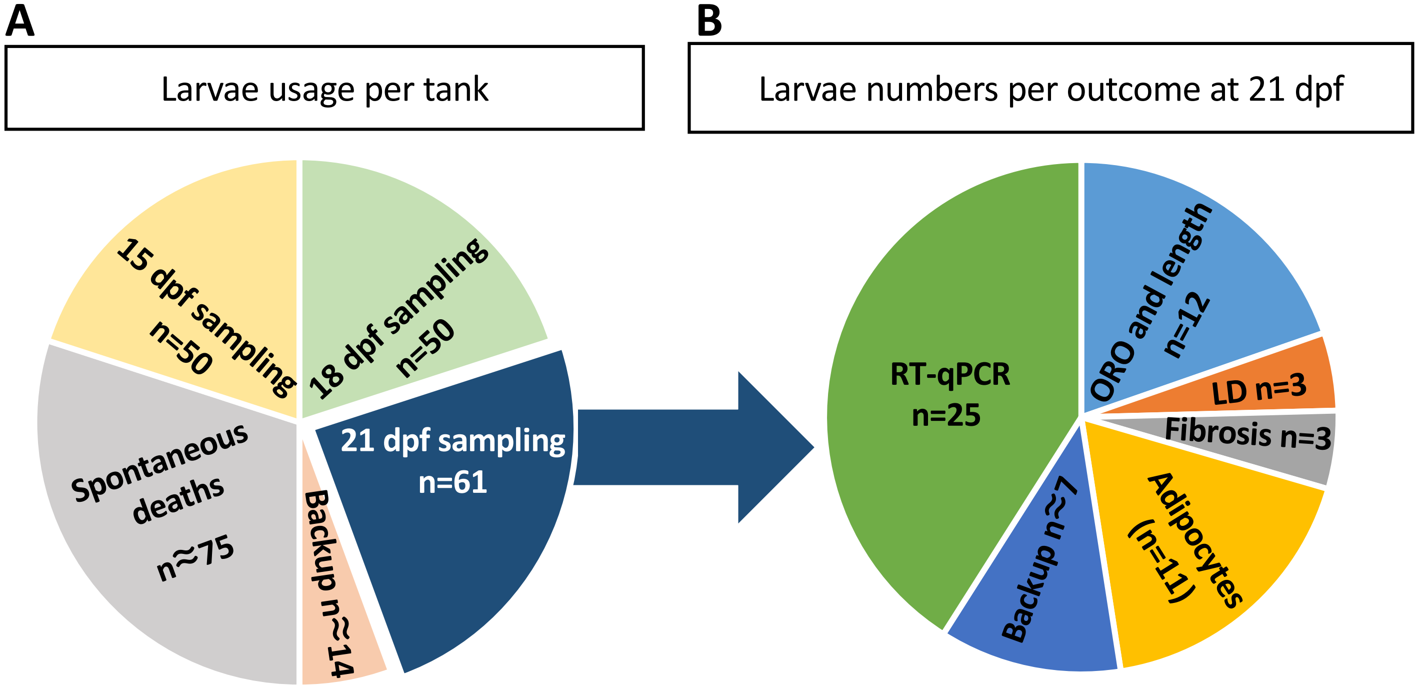

Supplemental figure 2: Representative images of lipid droplets volume quantification

A) Skeletal muscle from one 21 dpf larva per diet group with close up view of one of the four areas of interest used for quantification and 3D reconstruction. **B)** Liver from one 21dpf larva per group with close up view of one of the twelve areas of interest used for quantification and 3D reconstruction. For all panels, lipid droplets < $2um^3$ are in pink and > $2um^3$ in yellow.

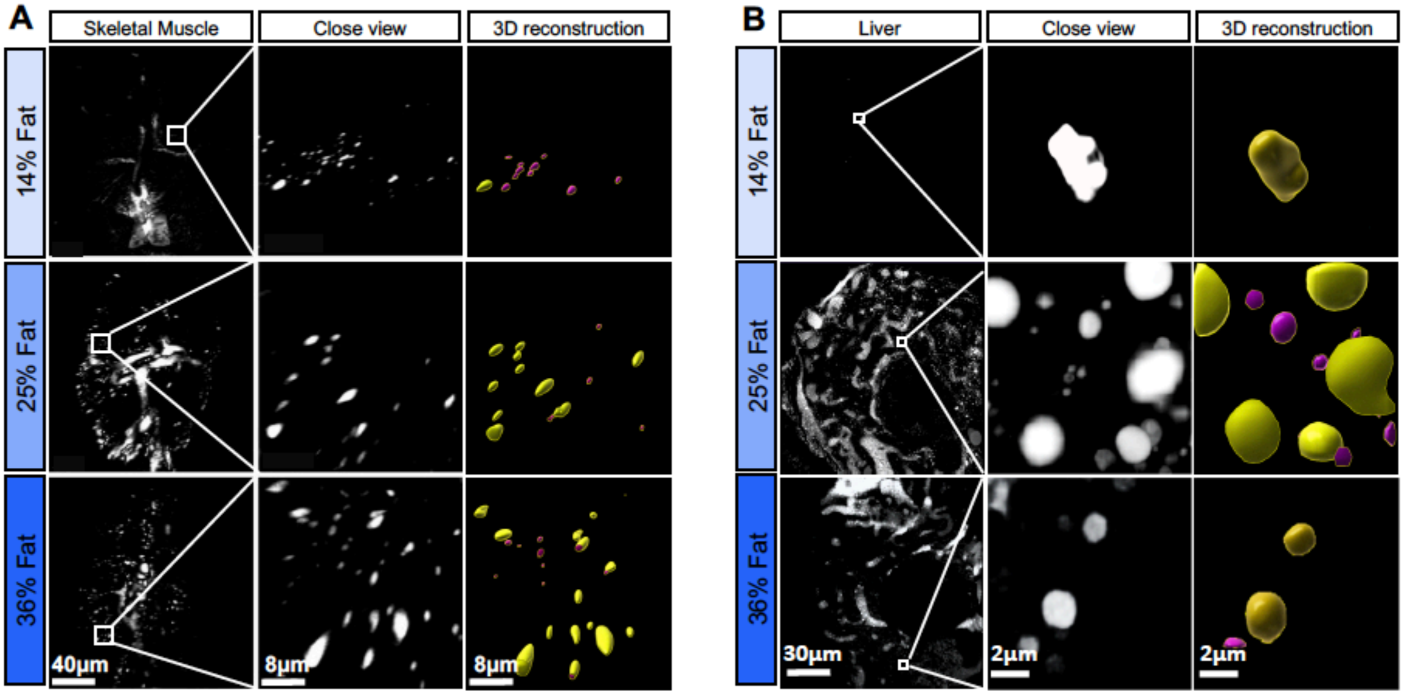

Supplemental table 1: Timing of experiments for embedding, staining, imaging and analyses

**Experiment**	**Approximate timing**	**Remarks**
Embedding	30 minutes	Medium needs to cool down to extract the blocks
Cryopreservation	12 hours	No active work
Cryosection	15 minutes / block	Depends on user experience
Staining	24 hours	Around 1 hour of active work, the rest are incubation times
Imaging	15 minutes / larva	Considering z-stacks and larva localisation in the slide
Quantification	15 minutes / larva	Depends on user experience

Supplemental table 2: Primers sequences

Target	Ensembl ID	Strand	Primer sequence
EF1α	ENSDARG00000020850	Forward	CCCCTGGACACAGAGACTTCATC
		Reverse	ATACCAGCCTCAAACTCACCGAC
ACCa	ENSDARG00000078512	Forward	GCGTGGCCGAACAATGGCAG
		Reverse	GCAGGTCCAGCTTCCCTGCG
FASN	ENSDARG00000087657	Forward	GGAGCAGGCTGCCTCTGTGC
		Reverse	TTGCGGCCTGTCCCACTCCT
LPL	ENSDARG00000087697	Forward	TACCTCAAAACCCGCGAGAT
		Reverse	TCGGCTGCTCCAGCAAAG
FABP11a	ENSDARG00000017299	Forward	AGACCACGACTGTCATGACTATCG
		Reverse	TTCCCATCCGACACCTCTCT
CPT1a	ENSDARG00000059770	Forward	CATCCTTAGGCCTGCTCTTCAAA
		Reverse	ACCATGACACCCCCAACTAACAT
CPT1b	ENSDARG00000058285	Forward	CCTCCATGGGCACGATTGATAA
		Reverse	GCAAACAGGATGGCACTCAACA
DGAT2	ENSDARG00000018846	Forward	TCATGGCTGTGGACTGTTTTTC
		Reverse	GAGTCGGCTCCTCTATCTTTGG
GPAT4	ENSDARG00000019897	Forward	GCCATCATCACCTACCATGACA
		Reverse	ACCTGACCGACCATTGCATAG
ATGL	ENSDARG00000089390	Forward	AACTCATCCAGGCTCTCAT
		Reverse	TTCCACCATCCACATAACG
HSL	ENSDARG00000101145	Forward	CGGCAAGGACAGGACAGT
		Reverse	GCATGGAGAAAGAGGAGCT
Seipin	ENSDARG00000037008	Forward	TCAGCAGAGACGAGAAGAAGCA
		Reverse	GAAGCGGATCTGAGAGTAGTTCACT
PLIN2	ENSDARG00000042332	Forward	CAGCTGCCTTTCACAGGTCTTT
		Reverse	TTTTCTATCTTATCCAGTCCTTTGCA
PLIN3	ENSDARG00000013711	Forward	GACAAGCTGGTATCCGACACAGT
		Reverse	TCTCCACACCCTCCATCACA
CIDEC	ENSDARG00000059651	Forward	GACTCAGCACTCCAGACC
		Reverse	TTCCAGTTCCATCCTCATCC
PPARa	ENSDARG00000031777	Forward	TGCTGGACTACCAGAACTGTGACA
		Reverse	TGCTGGCTGAGAACACTTCTGAG
PPARg	ENSDARG00000031848	Forward	AATTCGCCAAGAGCATCCCG
		Reverse	ATGAGCGGAGAAATCATGATGATC
FAF2	ENSDARG00000052374	Forward	TGGAGCGGAGATTCCTCTTC
		Reverse	CCGCGGGTAATTCGTCACTA
MGL	ENSDARG00000036820	Forward	ACGCTGACATTGCTCACAGTCT
		Reverse	TTGAGCTCCATCCTCTCACCTT
LDAH	ENSDARG00000079796	Forward	CAAACATTCTCCGCTCACAAAA
		Reverse	CTTCCGATCCTGAGCTGTTCA
METTL7A	ENSDARG00000056726	Forward	TCTTGAGCACGTGGTGTCAGA
		Reverse	TGAAAATCCTGCAGCGTCAAT
ACSL3	ENSDARG00000032079	Forward	TCTGGGATCACCGGAATGAC
		Reverse	CACACCATCTCAGCGCTCAA
